# Protective Role of *Phyllanthus niruri* Extract against Thioacetamide-Induced Liver Cirrhosis in Rat Model

**DOI:** 10.1155/2012/241583

**Published:** 2012-05-10

**Authors:** Zahra A. Amin, Mehmet Bilgen, Mohammed A. Alshawsh, Hapipah M. Ali, A. Hamid A. Hadi, Mahmood A. Abdulla

**Affiliations:** ^1^Department of Molecular Medicine, Faculty of Medicine, University of Malaya, 50603 Kuala Lumpur, Malaysia; ^2^Health and Translational Medicine, Faculty of Medicine, University of Malaya, 50603 Kuala Lumpur, Malaysia; ^3^Department of Chemistry, Faculty of Science, University of Malaya, 50603 Kuala Lumpur, Malaysia

## Abstract

A preclinical study was performed to determine if the extract from *Phyllanthus niruri* (PN) plays a protective role against liver cirrhosis induced by thioacetamide (TAA) in rats. Initially, acute toxicity was tested and the results showed that the extract was benign when applied to healthy rats. Next, the therapeutic effect of the extract was investigated using five groups of rats: control, TAA, silymarin, and PN high dose and low dose groups. Significant differences were observed between the TAA group and the other groups regarding body and liver weights, liver biochemical parameters, total antioxidant capacity, lipid peroxidation, and oxidative stress enzyme levels. Gross visualization indicated coarse granules on the surface of the hepatotoxic rats' livers, in contrast to the smoother surface in the livers of the silymarin and PN-treated rats. Histopathological analysis revealed necrosis, lymphocytes infiltration in the centrilobular region, and fibrous connective tissue proliferation in the livers of the hepatotoxic rats. But, the livers of the treated rats had comparatively minimal inflammation and normal lobular architecture. Silymarin and PN treatments effectively restored these measurements closer to their normal levels. Progression of liver cirrhosis induced by TAA in rats can be intervened using the PN extract and these effects are comparable to those of silymarin.

## 1. Introduction

Herbal products have long been used in traditional folk medicine to maintain health or to provide remedies for various human diseases [1]. Liver disorders, including liver cirrhosis, benefit from therapeutic strategies employing compounds extracted from plants and herbs [2]. *Phyllanthus niruri* (PN) is one of the herbal plants from the family of Euphorbiaceae and the extract from this plant was widely used in the preparation of various ayurvedic formulations [3]. The analysis of the extract revealed several bioactive molecules and chemical agents including phyllanthin, hypophyllanthin, phyltetralin, niranthin, nirtetralin, hinokinin and isolintetralin [4, 5]. Which are lignans belongs to the polyphenols group of compounds with well known antioxidant properties [6]. Application of the extract exhibited antiulcer, antitumor and anticarcinogenic, hypolipidemic, antiviral, and antioxidant effects. Past studies included the extract in a multiherbal preparation to treat liver ailments [7]. More recent explorations on the role of the extract has confirmed that it possesses potent protective effects against viral hepatitis and toxicity caused by different drugs or environmental toxicants [8].

In this study, the previously implicated hepatoprotective function of the PN extract was further evaluated and its efficacy as a therapeutic agent was experimentally tested on a rat model of liver cirrhosis induced chemically by thioacetamide (TAA) administration. TAA was originally used as a fungicide to preserve agricultural citrus products, but later it was determined that it was a potent hepatotoxin and carcinogen because its thono-sulfur-containing compound endowed with liver-damaging and carcinogenic activities [9]. On a prolonged exposure, TAA leads to the formation of hyperplastic nodules, cell adenomas, hepatocarcinomas, and cirrhosis. TAA-induced cirrhosis in rats has been shown to be a suitable experimental model of this disease with etiology and pathology comparable to the one seen in humans [10].

This study was performed as a continuum of the previous works reported by our laboratory Harish and Shivanandappa [7], or other groups Bhattacharjee and Sil [8], to objectively evaluate the therapeutic role of the PN extract on liver cirrhosis, we also employed another herbal substance silymarin with a well-established record of being a hepatoprotectant agent [11]. Silymarin is a purified extract obtained from the seeds of the plant *Silybum marinum* and used widely as a supportive therapy for liver disorders such as cirrhosis, hepatitis, and fatty acid infiltration due to alcohol and toxic chemicals [12]. The hepatoprotectant effect achieved with the PN extract was compared against the benchmark efficacy obtained from silymarin treatment. In the following, we describe each of the processes and procedures employed in our experiments, present results showing the changes in liver pathology and biochemicals with and without treatment in groups of experiments and discuss our findings in detail regarding the merits of the PN extract as a potential treatment agent against liver cirrhosis.

## 2. Materials and Methods

The study was approved by the institutional Ethics Committee, University of Malaya, Malaysia, with protocol number PM/28/08/2010/MAA. Throughout the experiments, we provided human care to the animals according to the criteria outlined in the “Guide for the Care and Use of laboratory Animals” prepared by the National Academy of Sciences and published by the National Institutes of Health.

Adult male healthy Sprague Dawley rats weighing between 190–260 g were acquired from the experimental animal house in our institute and were maintained on standard pellet diet and tap water and kept at 25 ± 3°C temperature, 50–60% humidity, and a 12 h light-dark cycle for at least one week before starting the experiment.

### 2.1. Preparation of the *Phyllanthus niruri* Extract

Fresh plants of PN were purchased from (Ethno Resources Sdn Bhd, Selangor Malaysia) and identified by comparison with the voucher specimen deposited at the Herbarium of Rimba Ilmu, Institute of Science Biology, University of Malaya, Kuala Lumpur (voucher number KLU46618). The plant was dried and grinded into fine powder. Hundred gram of the powder was soaked in 1000 mL of 95% ethanol for 3 days. Then, the mixture was filtered using filter paper (Whatman No. 1) and distilled under reduced pressure in a rotating evaporator (Buchi, Switzerland). The ethanol extract was then dissolved in 10% Tween 20 and administered orally to the rats.

### 2.2. Preparation of Thioacetamide

TAA (Sigma-Aldrich, Switzerland) was prepared freshly by dissolving in sterile distilled water (2 mL/kg body weight) and stirred well until all crystals were dissolved. Then, 200 mg/kg body weight was administered intraperitoneally (ip) to the rats thrice weekly for 8 weeks. The injection protocol above was according to the recommendation of Alshawsh et al. [2]. Constant exposure to a rat with this amount of TAA induces changes in its liver pathology from both biochemical and morphological aspects comparable to that of human liver cirrhosis [13] and therefore used very often as a preferred model in experimental studies of this disease.

### 2.3. Preparation of Silymarin

Silymarin (International Laboratory, USA) is a reference drug and (5 mL/kg body weight) was dissolved in sterile distilled water for oral administration to rats in doses of 50 mg/kg body weight.

### 2.4. Acute Toxicity Test

Tests were initially performed to determine the safety of the PN extract when administered orally, the acute toxicity test was performed following OECD-423 guidelines [14]. Twenty-four healthy Sprague Dawley rats (12 males and 12 females) were randomly assigned equally into 3 groups labeled as vehicle 10% Tween 20 and two large doses 2 g/kg and 5 g/kg of the extract, respectively. Each rat was made to fast (no food but water) overnight prior to dosing. Food was withheld for another 3 to 4 hours after the dosing. The rats were closely observed for 30 min and at 2, 4, 24, and 48 h after the dosing to detect if there were any acute signs of clinical or toxicological symptoms. After 14 days the rats were sacrificed to measure serum biochemical and (liver and kidney) histological parameters by following the standard methods [15].

### 2.5. Experimental Protocols

Forty male rats were randomly divided into five groups, each of which with eight rats: Group 1 (control group) rats were administered orally with 10% Tween 20 (5 mL/kg) daily and injected intraperitoneally with sterile distilled water (2 mL/kg) thrice weekly for 8 weeks. Group 2 (hepatotoxic group) rats were administered orally with 10% Tween 20 (5 mL/kg) and injected intraperitoneally (ip) with Thioacetamide (TAA) (200 mg/kg) thrice weekly for 8 weeks. The injection protocol above was according to the recommendation of Alshawsh et al. [2]. Group 3 (reference drug group) rats were administered orally with silymarin (50 mg/kg) daily and injected ip with TAA (200 mg/kg) thrice weekly for 8 weeks. Groups 4 and 5 (PN treatment groups) rats were administered orally with the PN extract 100 mg/kg and 200 mg/kg daily and injected ip with TAA (200 mg/kg) thrice weekly for 8 weeks [16].

In this regard, the experiment aimed to protect the liver from further deterioration. Body weights of rats in all groups were measured weekly. After 8 weeks, each rat was made to fast for 24 hours after the last treatment and then perfused under Ketamine (30 mg/kg, 100 mg/mL) and Xylazine (3 mg/kg, 100 mg/mL) anesthesia.

#### 2.5.1. Postmortem Tissue Analysis

The livers and spleens of the perfused rats were dissected, washed with normal saline, blotted with filter papers, weighed, and examined. The liver and spleen indices were calculated as the percentage of the body weight. Liver specimens were subsequently fixed in 10% buffered formalin and embedded in paraffin using automated tissue processing machine (Leica, Germany). Sections were sliced at 5 *μ*m thickness and stained with haematoxylin and eosin (H&E) for histological evaluation.

#### 2.5.2. Antioxidant Activity

Hepatic tissues from all livers were sampled from the same site of the left lobe, but away from the portal system. One gram of the sampled tissue was placed in 10 mL (10% w/v) of PBS (phosphate buffer solution with pH 7.4), then homogenized and centrifuged at 4000 rpm for 10 min at −4°C. The supernatant was kept in a −80°C freezer and assays were performed according to the instruction manual from the manufacturer. Commercially available kits (Cayman Chemical Co., USA) were used to determine the total antioxidant capacity (TAC) and malondialdehyde (MDA), catalase (CAT), superoxide dismutase (SOD), and glutathione peroxidase (GPX) content.

#### 2.5.3. Hepatic Biochemical Parameters

Blood of each rat was collected and serum was separated for analysis in the Clinical Diagnostic Laboratory (CDL) at University of Malaya Medical Center (UMMC) to determine the liver function enzymes such as alanine aminotransferase (ALT), aspartate aminotransferase (AST), alkaline phosphatase (ALP), bilirubin, albumin, total protein (TP), and gamma glutamyl transferase (GG).

### 2.6. Quantitative Estimation of Total Phenolic Contents

 The total phenolic content of PN was determined following the method of Basma et al. [17] with Folin-Ciocalteu reagent using gallic acid as standard. 10 *μ*L of extract solution (1 mg/mL) was added and followed by 0.5 mL of 1 : 10 Folin-Ciocalteu reagent. The mixture was incubated at room temperature for 5 minutes then 0.35 mL of 115 mg/mL natrium carbonate (Na_2_CO_3_) was added and mixed thoroughly. The mixture was then placed for 2 hrs at room temperature. Absorbance readings were taken spectrophotometrically at 765 nm. The total phenolic content was expressed in mg of gallic acid equivalents to gm PN extract.

### 2.7. Statistical Analysis

Statistical analysis was evaluated by using one-way analysis of variance (ANOVA) with posthoc test using Bonferroni multiple comparisons in the PASW program (version 18) for Windows (SPSS Inc. Chicago, IL, USA). The data were reported as the mean ± S.E.M.; a probability value less 0.05 was considered significant.

## 3. Results

### 3.1. Acute Toxicity Tests

There were no mortality in the rats administered with the doses 2 g/kg and 5 g/kg of the PN extract. Physically, they appeared normal and no signs of changes were observed in their skins, furs, eyes, and mucus membranes and salivations. Tremor, sleep, and behavior patterns were similar to those of the vehicle treated-rats. Their food intakes were normal and neither diarrhea nor other digestional problems was noticed. Weight gains in these rats paralleed to those in the vehicle group. The biochemical measurements reflected that these organs had normal functions, as supported by the quantitative data in Tables 1 and 2. Moreover, the vehicle group showed no abnormalities or side effects resulting from 10% Tween 20. These findings provided sufficient evidence to conclude that the orally administered extract was safe and did not cause extract-related toxicity.

### 3.2. PN Treatment Experiment

#### 3.2.1. Body, Liver, and Spleen Weights

The measurements of the body, liver, and spleen weights of the rats by the end of the study in all groups were listed in Table 3. The hepatotoxic rats weighted significantly less, but their livers and spleens weighted significantly more compared with the control rats. The rats subjected to the treatment with either silymarin or the PN extract exhibited gains in the body weight and losses in the liver and spleen masses, but the amounts were not as much as those in the control group.

#### 3.2.2. Gross Morphology

**Figure 1:**
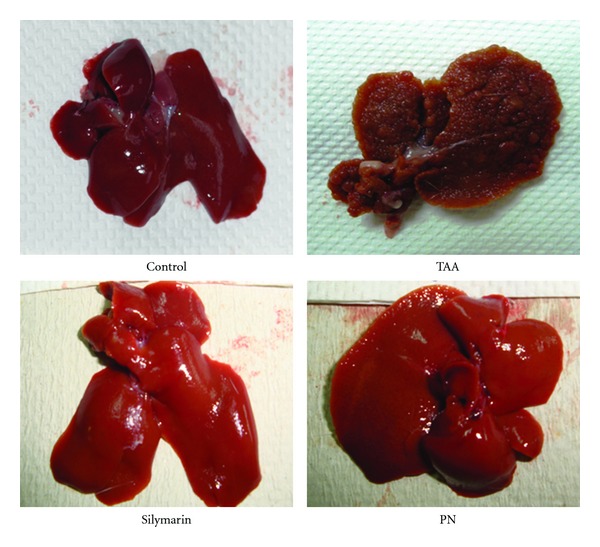
Images showing the macroscopic appearances of livers from different experimental groups. (Control): regular smooth surface. (Hepatotoxic): iirregular whitish micro- and macronodules and a large area of ductular cholangiocellular proliferation embedded within fibrosis. (Silymarin): smooth surface. (High dose PN): nearly smooth surface.

As depicted in Figure 1, the control rats had livers with smooth surfaces. In the hepatotoxic group, exposure to TAA made the liver attain irregular shape and at the same time be occupied with uniform formations of micronodules and macronodules (whitish granules), some of which were localized close to the surface. The nodular transformation in liver parenchyma is also a typical characteristic of human cirrhosis. The treatment with either silymarin or the PN extract prevented the development of the macro- and micronodules and made the liver preserve its nearly normal anatomical shape and appearance in rats. Although based on visual evaluations, these results clearly demonstrated that the PN extract effectively prevented the development of the nodules and hence protected the liver from further deterioration of its structure and function.

#### 3.2.3. Liver Histopathology

**Figure 2:**
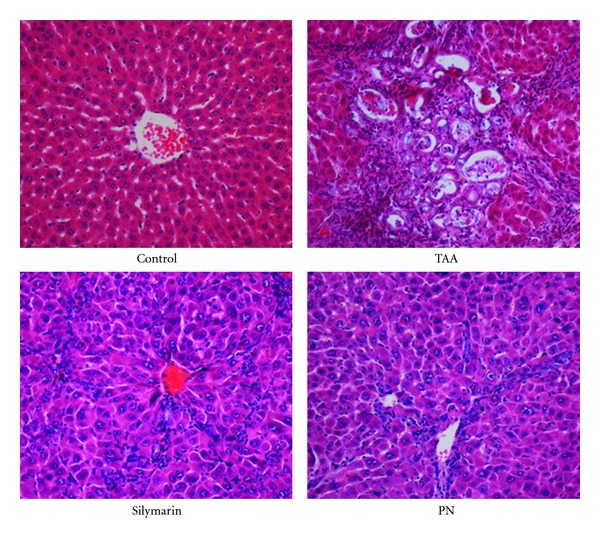
Histopathology images of the livers from different experimental groups. (Control): normal structure and architecture. (TAA): structural damage, necrosis, and pseudolobules with thick fibrotic septa. (Silymarin): mild inflammation but no fibrotic septa. (PN): partially preserved hepatocytes and architecture with small areas of mild necrosis.

Example slides were given in Figure 2. In the control group, the livers were clear of any pathological abnormality, and their sections looked normal with regular cellular architecture. The hepatic cells had intact cytoplasm, sinusoidal spaces, prominent nucleus, and nucleolus and central vein. In the hepatotoxic groups, the histology confirmed liver damage as evidenced by the presence of inflammation and necrosis in the respective liver sections. The architecture of the liver parenchyma was distorted by fibrous septa that formed collagen bridges between the hepatic triads delineating the small and large regenerative nodules of hepatocytes or necrosis. The nodules were surrounded by a thick fibrous septa which divided the liver into pseudolobules. There were observations of cytoplasmic vacuolization, megalocytosis, bile duct proliferation, hepatocyte degeneration, necrosis, infiltration of inflammatory cells, and centrilobular necrosis with portal triaditis, expanded portal tracts, and collagen deposition. The spindle and hepatic cells surrounding the central vein proliferated and various degenerative changes like cloudy swelling, hydropic degeneration, loss of nucleus and nucleolus, and necrosis have taken place [18].

The rats in silymarin-treated group exhibited significantly lesser pathology as compared to the extensive liver damage found in the hepatotoxic group. Silymarin administration prevented the swelling, lymphocytes infiltration, hepatic necrosis, and fibrous connective tissue proliferation induced by TAA. Consequently, the liver tissue preserved its nearly normal hepatic lobular architecture with central veins and radiating hepatic cords. These results reconfirmed the protective functions of silymarin against the TAA-induced liver damage.

The histopathological examination of the liver sections from the rats treated with the PN extract revealed reduced degrees of fibrosis, but smaller necrotic zones with nodules and centrolobular veins still occupied the parenchyma. The livers had appreciable levels of normal parenchymal architecture with thinner fibrous septa, and lesser amounts of cytoplasmic vacuolization, megalocytosis, bile duct proliferation, and nucleic injury. These results provided microscopic evidence that demonstrated the significant hepatopreservation function of the PN extract to counterbalance the negative effects of TAA in the liver tissue.

#### 3.2.4. Antioxidant Activity

**Figure 3:**
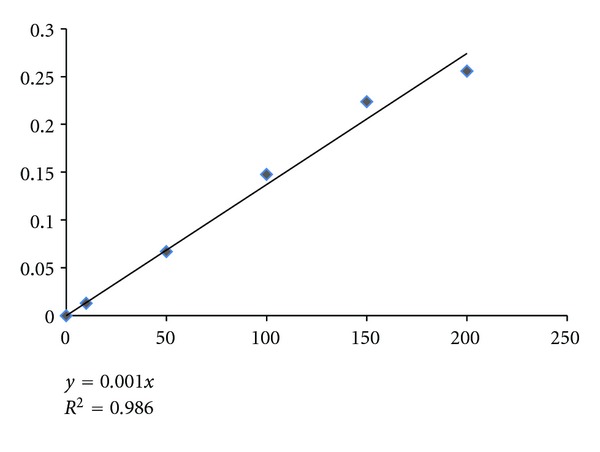
Standard curve of quantitative estimation of total phenolic content in PN extract.

Quantitative estimation of total phenolic contents of PN revealed that this plant enclose (270 ± 0.003 mg GAE/gm of PN extract) polyphenolic compounds with standard curve equation shown in Figure 3.

Moreover, Table 4 lists the activities of enzymes involved in the hepatic antioxidant defense system. It is recorded that SOD, CAT, and GPX enzyme levels were increased in TAA groups when compared with the control groups and similarly their levels decreased in silymarin and the PN extract treated groups. Additionally, the total antioxidant capacity (TAC) values were substantially increased in the treatment groups in comparison to the TAA group. However, data from Table 4 indicates that the lipid peroxidation values in the livers of the PN treated rats were lower than those in the TAA group. This set of data indicates that the PN extract is an antioxidant and protects the liver through this property.

#### 3.2.5. Hepatic Biochemical Parameters

The activities of alkaline phosphatase (ALP), alanine aminotransferase (ALT), aspartate aminotransferase (AST), bilirubin, total protein, albumin, and gammaglutamyl transferase (GG) were shown in Table 5. Significant increases in the serum ALP, ALT, AST, bilirubin, and GG, but substantially lower total protein and albumin levels were detected in the hepatotoxic rats compared to those in the control group. Silymarin therapy was effective and restored the activities of these serum biochemicals closer to their normal levels. Treatments with the PN extract at the dose 200 mg/kg produced activities similar to those seen with silymarin. These results demonstrated the capacity of the PN extract to control the abnormal hepatic biochemical activities initiated by TAA.

## 4. Discussion

 Liver functions as an endogeneous metabolism center for nutrients, such as carbohydrates, proteins, and lipids, and also participates in disposal of waste metabolites. The organ also handles the metabolization or excretion of exogeneous drugs and other xenobiotics. In this regard, liver plays a major role in protecting and detoxifying the body from foreign substances [19]. Liver cirrhosis is a major disease associated with various pathological processes including progressive fibrosis, portal hypertension and carcinoma [20]. Free radical generation, mitochondrial dysfunction and depletion of antioxidants lead to the progression of fibrosis and cirrhosis [21]. Thioacetamide is an organic solvent with thiono-sulfur components have been used widely to induce liver cirrhosis [22–26]. In the present study, the prolonged administration of TAA to the rats caused visual and quantifiable responses, which were recognizable by the alterations in the body and liver weights, appearance of gross morphology, distinct stained patterns of histopathology, and levels of serum molecular markers. All these indications were of cirrhosis and supported the past reports that TAA contributes to the development of cirrhosis through multiple mechanisms of action [27] like the oxidation of its metabolic products [28, 29], oxidative stress [30, 31], and decreased antioxidant defenses and lipid peroxidation. During the eight-week-long study, the hepatotoxic rats lacked in gaining body weight as compared with the controls (Table 3). Using the same experimental model of cirrhosis, the previous studies reported the same and attributed this outcome to the lower levels of nutrient absorption, energy utilization, and metabolic efficiency as the major factors affecting the inability of the rats to gain weight after being exposed to TAA [2]. Factoring the reduced body weight into the calculation yielded significantly high ratios of liver and spleen-to-body weight. The hepatocyte proliferation is a critical determinant for the survival of liver from an injury [32]. Based on this, the upregulation of the hepatocyte activity in response to the exposure to TAA toxicity is likely to be the cause of the recorded increase in the liver and spleen weights.

Ex vivo examinations of the livers from the hepatotoxic rats exhibited numerous white and yellow-colored nodules (Figure 1), which closely resembled the human cirrhosis with hepatocarcinoma in both pathobiological and morphological aspects [10]. The agreement between our results and those of the previous works in several fronts confirmed that our experimental rat model of cirrhosis was suitable for testing the efficacy of any applied preclinical therapy with a clinical translation in focus.

This study showed that liver's total antioxidant capacity in vivo (TAC) reduced in the TAA-treated rats when compared to control rats while the PN and silymarin treatments in turn elevated the levels near to control. On the other hand, TAA group exhibited higher values of cellular lipid peroxidation which measured through the malonialdehyde level (MDA) than did the control livers. SOD, CAT, and GPX levels in the liver homogenates of PN-treated rats were found to be significantly higher than the TAA group. These results are in agreement with the previous studies which reported that the reason of PN's usage in treatment of liver diseases is in its efficacy in inhibition of reactive oxygen species and lipid peroxidation [33].

In the hepatotoxic rats, we detected elevated levels of serum ALT, AST, ALP, and total bilirubin concentrations (Table 5), which were typically measured for assessing the liver function. Such enzymatic activities were in line with the earlier reports [32]. The increase in serum enzymatic activities is related to hepatic parenchymal damage since ALT is released from mitochondrial and cytosolic localization from membranal sites, and cellular rupture allows the enzyme to escape into the blood [33]. The raised serum liver enzymes such as ALT, AST, and ALP in intoxicated rats compared to normal indicates necrosis of hepatocytes that results in the leakage of transaminase and the elevation of serum ALP from a possible cholestasis and this also can be attributed to the damage in the histostructural integrity of the hepatocytes [34].

TAA grossly impairs the erythrocyte plasma membrane stability, producing a labile membrane which was easier to lyse. High dose of bilirubin detected in the hepatotoxic rats was an indication of the increased erythrocyte degeneration rate. The liver excretes the breakdown products of hemoglobin, particularly bilirubin, whose level is used to evaluate chemically induced hepatic injury and hence reflect the necrotic conditions of hepatocytes [33]. The total protein and albumin levels are depressed in hepatotoxic conditions due to disturbances in the carbohydrate, protein, lipid metabolisms or perturbed protein biosynthesis in the cirrhotic liver. The marked reduction of the total protein synthesis in the hepatotoxic rats in our experiments was in agreement with the early observations of decreased ribosomal RNA in TAA-treated rats cells. Since the decrease in RNA occurs in cells where RNA polymerases is activated, defective intranuclear RNA processing is the most likely explanation of the observed decrease [35].

Silymarin is a well-established plant-based formulation and a clinical drug with proven capacity to guard liver from harmful hepatotoxins. Such pharmacological power was attributed to silymarin's inherent constituents with antioxidant, anti-inflammatory, and diuretic properties, as in other medicinal plants in nature [11]. In addition, silymarin has the capacity as antilipid peroxidation and induced detoxification system, protector of cell against employed glutathione, reducer of leukotiene formation from unsaturated free acid, enhancer of protein synthesis, stabilizer of mast cells and regulator of immune functions. It inhibits cytoP450 detoxification system and prevents metabolism of toxic compound such as TAA [36].

In our study, we reconfirmed that silymarin played substantial role against the progression of cirrhosis induced by TAA in rats (Figures 1 and 2 and Tables 3, 4, and 5). It significantly reduced the liver pathology indicated by the declined levels of ALT, AST, ALP, bilirubin, and increased albumin and total protein levels activities as compared to the hepatotoxic rats. The readings for these biochemicals were the best achievable values and therefore set the baseline for comparisons of the therapeutic improvements attained with the PN extract.

The treatment of the rats with the ethanol extract from the plant PN has proven to be a useful strategy against the pathophysiological and biochemical changes induced by the hepatotoxin TAA in the rat model of cirrhosis (Figures 1 and 2 and Tables 3, 4, and 5). We however note that the PN extract reversed the toxicity produced by TAA in a dose dependent manner, where 200 mg/kg was more effective than 100 mg/kg of dose. Nevertheless, in the rats treated with PN, the bodies weighted more and livers and spleens weighted less compared with those of the cirrhotic rats. The treatment ultimately resulted in decreased liver to body weight ratio, which was closer to that of the controls (Table 3). Visual evaluation and histopathological analysis demonstrated that the liver structure and architecture remained nearly intact and contained lesser fibrosis and lower number of nodules than the cirrhotic rats (Figure 2). Biochemical analysis indicated that parameters of interest read closer to the levels measured from the control rats. The data in Table 5 indicates that the extract treated the rise of the serum levels of ALT, AST, ALP, and bilirubin, and the decline in the levels of albumin and total protein. The reduction seen in the levels of these enzymes in the treated rats hinted that the PN extract has stabilized the hepatocytes membranes and interrupted the release of enzymes from liver into blood. The lowered bilirubin levels supported this action since it implied more stable erythrocyte plasma membranes were present in the treated rats. These findings are consistent with those of Harish and Shivanandappa [7] who found that aqueous and methanolic extracts of *P. niruri* showed inhibition of membrane lipid peroxidation, scavenging of DPPH (1,1-diphenyl-2 picrylhydrazyl) radical and inhibition of reactive oxygen species in vitro. Also pretreatment with PN inhibits carbon tetrachloride (CCl_4_) induced formation of lipid peroxides in the liver of rats in vivo. Moreover Sabir and Rocha [37] demonstrated the hepatoprotective activity of PN extract in vivo against paracetamol-induced liver damage. While Chatterjee and Sil [38] results suggested that beneficial effect of the aqueous extract of PN, probably through its antioxidant property, might control the nimesulide-induced oxidative stress in rats liver.

Thioacetamide is a well-known liver hepatotoxicant, the hepatotoxicity results from its metabolic conversion to free radical products: thioacetamide sulfoxide and thioacetamide-S, S-dioxide which attacks microsomal lipids leading to their peroxidation, and production of reactive oxygen species (ROS), such as the H_2_O_2,_ super oxide anion O_2_^−^ and the hydroxyl radical. ROS affects the antioxidant defense mechanisms, decreases the activity of SOD, CAT, and GPX that causes liver injury, cirrhosis development, and hepatocarcinoma [39]. Nevertheless, plant derived polyphenols were well-known free radical scavengers and effective as antioxidants against lipid peroxyl radicals [40]. Since PN enclosed total phenolic content of 270 ± 0.003 mg GAE/gm of PN extract, thus, the protective activity of PN may be due to its polyphenols active ingredients; phyllanthin, hypophyllanthin, phyltetralin, niranthin, nirtetralin, hinokinin, and isolintetralin that identified to exhibit many biological activities, such as scavenging of free radicals [6, 41] and inhibiting lipid peroxidation [42], which are TAA's well-known mechanisms of action in producing liver injury. The capacity of the PN extract to regulate the hepatic antioxidant status or to directly participate in H_2_O_2_, super oxide anion O_2_^−^, and the hydroxyl radical scavenging process explains the trends in the measurements towards restoring the balance in serum chemicals [43].

## 5. Conclusions

All the observations made and measurements collected in this study provided preliminary evidence that the progression of the liver cirrhosis induced by TAA in rats can be intervened using the PN extract. Specifically, this natural extract has power to protect the liver by preventing the actions of the harmful events associated with the TAA toxicity from taking place. The effects are comparable to those of silymarin and the capability of the PN extract to preserve the liver's status quo of property, structure, and function against toxic exposure is encouraging and warrants further studies exploring the significance of its pharmacologic potential in treating the liver cirrhosis by mapping the molecular pathways of action.

## Figures and Tables

**Table 1:** Effects of 2 g/kg and 5 g/kg PN ethanol extract on liver biochemical parameters.

Groups	ALT (IU/L)	AST (IU/L)	ALP (IU/L)	Total protein (g/L)	Albumin (g/L)	Total bilirubin (*μ*mol/L)	G-glutamyl transferase (IU/L)
Tween 20 (10%)	45.00 ± 5.32	27.00 ± 4.16	97.67 ± 13.86	73.00 ± 2.96	44.17 ± 2.59	10.17 ± 2.26	58.17 ± 9.90
PN extract (2 g/kg)	49.67 ± 4.60	26.33 ± 3.22	92.67 ± 9.76	74.50 ± 2.31	42.83 ± 1.97	8.00 ± 1.06	37.67 ± 6.82
PN extract (5 g/kg)	45.17 ± 5.30	28.00 ± 3.65	78.33 ± 6.81	74.33 ± 2.72	42.50 ± 2.93	9.50 ± 1.69	55.00 ± 8.83

Values were expressed as mean ± S.E.M. There are no statistically significant differences between the measurements in different groups. The significant value was set at *P* < 0.05.

**Table 2:** Effects of 2 g/kg and 5 g/kg PN ethanol extract on kidney biochemical parameters.

Groups	Sodium (mmol/L)	Potassium (mmol/L)	Chloride (mmol/L)	CO_2_ (mmol/L)	Anion gap (mmol/L)	Urea (mmol/L)	Creatinine (*μ*mol/L)
Tween 20 (10%)	141.33 ± 1.59	4.33 ± 0.27	102.83 ± 1.05	26.83 ± 1.72	14.33 ± 1.75	4.60 ± 0.70	37.33 ± 0.18
PN extract (2 g/kg)	140.00 ± 1.77	4.37 ± 0.30	103.17 ± 1.45	26.33 ± 1.59	13.00 ± 1.44	4.57 ± 0.70	37.60 ± 0.19
PN extract (5 g/kg)	137.17 ± 1.14	4.42 ± 2.39	102.67 ± 0.99	22.17 ± 0.60	17.83 ± 1.45	4.73 ± 0.67	37.52 ± 0.11

Values were expressed as mean ± S.E.M. There are no statistically significant differences between the measurements in different groups. The significant value was set at *P* < 0.05.

**Table 3:** Body, liver, and spleen weights of the rats in different groups.

Groups	Body wt. (gm)	Liver wt. (gm)	Liver index (%)	Spleen wt. (gm)	Spleen index (%)
Control	237.0 ± 17.24	6.2 ± 0.49	2.63 ± 0.13	0.36 ± 0.02	0.16 ± 0.02
TAA	195.4 ± 18.35	9.4 ± 0.98	4.81 ± 0.23^a^	0.51 ± 0.10	0.25 ± 0.04
Silymarin	222.0 ± 12.00	9.2 ± 0.97	4.10 ± 0.22	0.53 ± 0.04	0.24 ± 0.01
PN 100 mg/kg	258.6 ± 14.02	8.2 ± 1.02	3.22 ± 0.41^b^	0.46 ± 0.04	0.18 ± 0.02
PN 200 mg/kg	253.4 ± 16.41	7.8 ± 0.58	3.18 ± 0.24^c^	0.40 ± 0.04	0.16 ± 0.01

The data were stated as mean ± S.E.M. Means with different superscripts are significantly different. ^a^ *P* < 0.05 versus normal control group, ^b^ *P* < 0.05 versus TAA control group, and ^c^ *P* < .01 versus TAA control group.

**Table 4:** Effects of TAA, PN, and silymarin on antioxidant enzymes activities.

Groups	TAC (mM)	CAT (nmol/min/mL)	SOD (U/mL)	MDA (*μ*M)	GPX (nmol/min/mL)
Control	7.83 ± 1.47	848.3 ± 2.11	246.6 ± 4.12	30.10 ± 9.09	1438.4 ± 1.4
TAA	4.48 ± 1.54	964.9 ± 4.5^a^	310.3 ± 10.0^a^	59.77 ± 8.41^a^	1466.1 ± 2.0
Silymarin	8.37 ± 0.43^b^	839.7 ± 48.3^c^	280.2 ± 12.3	30.43 ± 2.53^c^	1437.8 ± 55.9
PN 100 mg/kg	6.73 ± 0.21	898.3 ± 1.9	293.6 ± 29.2	37.77 ± 8.35^b^	1457.0 ± 36.1
PN 200 mg/kg	6.72 ± 0.91	854.03 ± 15.9^c^	287.8 ± 5.55	29.10 ± 8.25^c^	1451.2 ± 9.4

The data were stated as mean ± S.D. Means with different superscripts are significantly different. ^a^ *P* < 0.05 versus normal control group, ^b^ *P* < 0.05 versus TAA control group, and ^c^ *P* < 0.01 versus TAA control group.

**Table 5:** Effects of TAA, PN, and silymarin on hepatic biochemical parameters.

Groups	ALT (IU/L)	AST (IU/L)	ALP (IU/L)	T. protein (g/L)	Bilirubin (*μ*mol/L)	Albumin (g/L)	GGT (IU/L)
Control	66.0 ± 3.63	162.6 ± 9.35	109.8 ± 10.85	72.0 ± 1.16	2.0 ± 0.00	11.0 ± 0.32	8.4 ± 0.40
TAA	165.6 ± 19.58^a^	484.8 ± 103.83^a^	431.2 ± 72.94^a^	58.80 ± 1.07^a^	7.4 ± 1.07^a^	8.4 ± 0.22	18.4 ± 3.5^a^
Silymarin	63.0 ± 4.16^c^	158.0 ± 16.77^c^	86.0 ± 51.45	67.20 ± 2.73^c^	1.8 ± 0.32^c^	10.8 ± 0.73	13.3 ± 0.97
PN 100 mg/kg	82.6 ± 4.10^c^	282.2 ± 24.57^c^	272.4 ± 17.38^c^	70.0 ± 1.00^b^	5.2 ± 0.92	10.4 ± 0.38	15.6 ± 2.25
PN 200 mg/kg	78.2 ± 6.39^c^	180.6 ± 25.86^c^	140.2 ± 26.63^c^	71.25 ± 0.66^c^	3.4 ± 0.37^c^	10.7 ± 0.15	11.0 ± 1.76

All values are expressed as mean ± S.E.M. Means with different superscripts are significantly different. ^a^ *P* < 0.05 versus normal control group, ^b^ *P* < 0.05 versus TAA control group, and ^c^ *P* < 0.01 versus TAA control group. ALT: alanine aminotransferase, AST: aspartate aminotransferase, ALP: alkaline phosphatase, GGT: gamma glutamyl transferase.
